# Record-Breaking Rain Event Altered Estuarine Viral Assemblages

**DOI:** 10.3390/microorganisms10040729

**Published:** 2022-03-29

**Authors:** Alaina C. Woods, Jordan R. Walker, Cameron D. Jackson, Jessica M. Labonté

**Affiliations:** Department of Marine Biology, Texas A&M University at Galveston, Galveston, TX 77553, USA; alaina.woods@maine.edu (A.C.W.); jronwalker@tamu.edu (J.R.W.); cjacks89@vols.utk.edu (C.D.J.)

**Keywords:** viral ecology, hurricane, metagenomics, auxiliary metabolic genes

## Abstract

Viruses are the dominant biological entity in the ocean, play a vital role in biogeochemical cycles, and provide their hosts with novel metabolic capabilities through auxiliary metabolic genes (AMGs). Hurricane Harvey was a category 4 hurricane that made landfall on the Texas coast in 2017 and lashed the Houston area with 1.4–1.7 × 10^10^ m^3^ of rainfall. In this paper, we aim to characterize how the changes in abiotic conditions brought by Hurricane Harvey altered the viral assemblages of Galveston Bay at the taxonomic level and determine how viral ecosystem functions were altered. Metagenomes of the viruses and their hosts were sequenced from a transect in Galveston Bay over the five weeks following the storm. Our results show that the viral assemblages of Galveston Bay dramatically changed following Hurricane Harvey’s landfall. Of the abiotic parameters measured, salinity had the strongest effect on shaping the viral assemblages. In the five weeks following Hurricane Harvey, there was a steady increase of metabolic genes and putative viral infections. Our study provides the first in-depth look at how marine viral assemblages respond and recover from extreme rainfall events, which models predict will become more frequent and intense with climate change.

## 1. Introduction

Viruses are the dominant biological entity in the ocean, with their numbers estimated to be as high as ~4 × 10^30^ [1]. They play many important roles in aquatic ecosystems, including top-down control, nutrient recycling, and increasing the fitness of the host in unfavorable conditions [2,3]. Viral infections cause the lysis of an estimated ~10–20% of the marine prokaryotic population daily [1,4,5], and the ecosystem-wide effects of viral lysis can alter geochemical cycles in the ocean by redirecting nutrients from higher trophic levels back to the microbial food web as dissolved organic matter, a process called the viral shunt [6,7,8,9,10,11]. Viruses are important in directing carbon and energy flux in marine ecosystems.

Auxiliary metabolic genes (AMGs) are viral encoded genes that typically originate from their hosts, with metabolic capabilities that can redirect energy and resources, improving viral production within the host [12]. In surface ocean environments, some cyanophages (viruses infecting Cyanobacteria) encode photosynthetic genes [13], and nearly 40% of sequenced phage genomes encode for the phosphate regulation uptake gene, phoH [14]. At least 31 additional AMGs have been identified in cyanophages, and phylogenetic analyses revealed that rare AMGs are more frequently gained or lost as a result of fluctuating selection pressures, while common AMGs are associated with stable selection pressures [15]. As the viral genomic diversity is revealed, more AMGs are discovered. For example, among the viral genomes assembled from the *Tara* Oceans and Malaspina research expeditions, a total of 243 virally-encoded AMGs were identified, comprising nearly 150 genes not previously known as viral AMGs [7]. With AMGs, viruses may directly manipulate the biogeochemical cycles such as those of sulfur, nitrogen, and phosphorus [7], although more studies are necessary to quantify the impact of viral AMGs within these cycles. The presence of AMGs in viral genomes and potential transfer of AMGs between hosts and viruses or viruses and their hosts may provide greater fitness to the microbial communities in the face of unfavorable conditions [2], such as those brought by a large-scale rain event.

While it has been well documented that microbial communities respond to pulse disturbances such as hurricanes [16,17] and oil spills [18], there has been little work done on the impact of pulse disturbances and large rain events on viral assemblages. After Hurricane Sandy, Williamson et al. [19] evaluated the impact of stormwater runoff on the viral community composition in a freshwater system and found that the storm had negative impacts on viral species richness and viral abundances due to increased rainfall and bacterial abundance. In contrast, another study used metagenomics to study the impact of a large rain event on the viral assemblages of a retention pond and found that following the storm, stormwater runoff contributed to a sharp increase in viral richness and diversity [20]. In this study, we looked at the impact of a pulse disturbance, Hurricane Harvey, on the viral communities inhabiting the Galveston Bay estuary. Hurricane Harvey made landfall near Port Aransas, TX, on 25 August 2017. Galveston, TX, located ~280 km northeast of Port Aransas, did not experience hurricane-force winds but experienced a tropical storm with winds between 60–120 km/h and a storm surge of <1 m. Moreover, Hurricane Harvey stalled over Houston and Galveston Bay, causing a record-breaking 1.4–1.7 × 10^10^ m^3^ of rainfall to lash the area over the next four days (25–29 August 2017). Floodwater, stormwater, and rainwater all flushed into Galveston Bay, causing a drop in salinity, temperature, and dissolved oxygen [17,21,22] (Supplementary Table S1). Moreover, 9.86 × 10^7^ metric tons of sediment were resuspended into Galveston Bay, causing an increase in dissolved organic matter, nutrients, and turbidity [21] (Supplementary Table S1).

To characterize the impact of Hurricane Harvey on the viral assemblages, we sequenced viral and microbial metagenomes from a transect in Galveston Bay for five weeks following the storm. We characterized how the change in environmental conditions changed the viral assemblages at the taxonomic level. We hypothesized there would be an increase in viruses associated with the terrestrial environment in Galveston Bay following Hurricane Harvey. We also identified how the storm impacted viral ecosystem functions through the identification of AMGs and estimation of putative viral infections. We hypothesized that viral function would change due to the changing environmental conditions [17,21,22] and host community [17], and there would be a decrease in putative viral infections due to the excess runoff [21], diluting viral concentrations and reducing virus–host contacts. Our results show that the viral assemblages changed after the storm and returned to estuarine viruses as the environmental conditions (i.e., salinity and temperature) returned to pre-storm levels. There was also a constant increase of metabolic genes and putative viral infections over the following five weeks, suggesting that the storm’s long-term impact on viruses resulted in accelerated geochemical cycles.

## 2. Materials and Methods

### 2.1. Sample Collection and Processing

**Figure 1:**
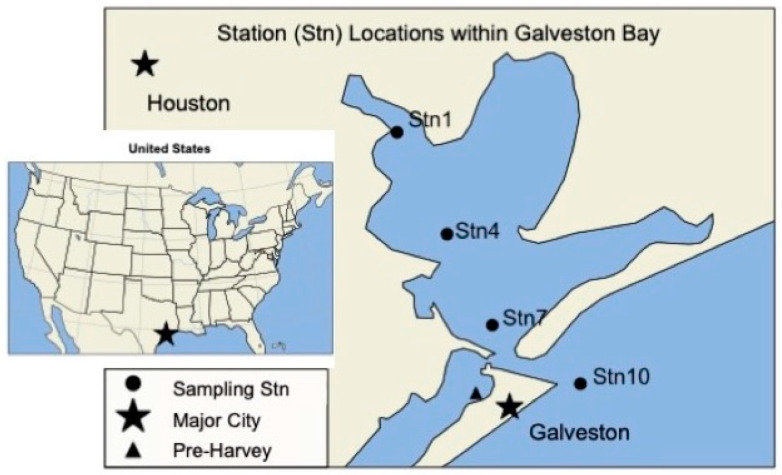
Sampling map of Galveston Bay, Texas (United States) showing the sampling stations used during each of the sampling efforts. The triangle represents the Texas A&M University at Galveston boat basin, where pre-Harvey samples were taken on 31 July and 22 August 2017.

Samples were collected from four stations in Galveston Bay after Hurricane Harvey left the Galveston Bay area on 4 September, 9 September, 16 September, and 28 September 2017 onboard the Texas A&M University at Galveston (TAMUG) vessel R/V Trident. A four-station transect (Stn1 (−94.976944, 29.6725), Stn4 (−94.897222, 29.535556), Stn7 (−94.825833, 29.4125), and Stn10 (−94.688611, 29.333611)) was sampled from the mouth of the San Jacinto River to the Gulf of Mexico (Figure 1). Sampling trips started in the morning and were always transiting from Stn1 to Stn10. Pre-Harvey samples were collected from TAMUG’s boat basin (−94.85, 29.32) on 31 July and 22 August 2017 prior to Hurricane Harvey’s landfall. The pre-Harvey samples are geographically closest to Stn7 but are not directly in Galveston Bay. The environmental conditions at the TAMUG Boat Basin are more closely related to those found at Stn10, at the mouth of the Gulf of Mexico (Supplementary Table S1). Due to the possible differences in environmental parameters, we will compare the post-Harvey samples to the pre-Harvey samples but refrain from making assumptions regarding ecosystem recovery. For this study, 20–25 L of surface water was collected using a 5-gallon bucket attached to a rope. All samples were pre-filtered onboard immediately after sampling with a Nitex filter (30 μm) to remove small grazers and large particles. After pre-filtration, the samples were stored on the boat in the dark at room temperature for up to 4 h in HDPE carboys and brought to the laboratory, where they were stored at 4 °C until filtration. Filtration always occurred on the same day as sampling. Generally, each sample was filtered through a 142 mm glass fiber filter (Whatman GF/F with a 0.7 μm pore-size or Whatman GF/D with a 2.7 μm pore-size), followed by a 142 mm 0.22 μm pore-size polyvinylidene fluoride (PVDF) filter (Supplementary Table S2 details which filter was used for each sample). Due to the availability of supplies, for the sampling of 9 September, pre-filtration was performed with a 142 mm 0.45 μm filter and the virus concentrate was then filtered through 47 mm 0.22 μm PVDF filter. The volume of sample filtered ranged from 4 to 20 L, depending on time constraints, available material, and personnel (Supplementary Table S2). All GF and PVDF filters were stored at −20 °C until further use. Filtered samples, which contained the viruses, were stored at 4 °C until virus concentration, for no more than 24 h. Viruses were concentrated using tangential flow filtration with a 30 kDa cut-off as previously described [23] (Supplementary Table S2). All virus concentrates were stored at 4 °C until DNA extractions.

### 2.2. DNA Extractions and Sequencing 

Viral DNA was extracted from the virus concentrates corresponding to an initial volume of 4 L estimated from the concentrated final volume and concentration factor (Supplementary Table S2). Before extraction, virus concentrates were further concentrated to a final volume of ~500 μL using 30 kDa Amicon Centrifugal Filters (Millipore, Burlington, MA, USA). Viral DNA was extracted using the DNeasy PowerSoil DNA extraction kit (Qiagen, Germantown, MD, USA). Since the yield was too low for metagenomic sequencing, the DNA was amplified using the Repli-g DNA Mini kit (Qiagen) according to the manufacturer’s recommendations. The amplified DNA was further purified with the QIAamp DNA purification kit (Qiagen) following the cleanup of genomic DNA protocol. Total DNA from the putative host fraction was extracted from the GF/F, GF/D, and 0.22 µm filters to the corresponding volume of 4 L using a standard phenol-chloroform extraction protocol [24], where the lysis buffer consisted of 400 mM NaCl, 750 mM sucrose, 20 mM EDTA, and 50 mM Tris-HCl, pH = 9.0. All DNA extracts were quantified with a QuBit fluorometer [with the HS dsDNA Assay kit (Invitrogen, Waltham, MA, USA)] and stored at −20 °C until further use. Between 1–10 μg of DNA from each sample were sent for sequencing using Illumina HiSeq 4000 with 150 bp paired-end sequencing technologies at the Texas A&M Genomics and Bioinformatics facility (College Station, TX, USA).

### 2.3. Metagenomic Data Analysis 

BBtools software suite (version 38.31; available at https://sourceforge.net/projects/bbtools/ (accessed on 1 June 2019)) was used for quality control of the metagenomic raw reads [25], as follows. BBduk was run twice, once to remove contaminants (kmers between 11–23 bp for common adapter artifacts) and once to trim reads that contained adapter sequences and had low quality (Phred score Q < 10). BBmerge was used to merge the forward and reverse paired-end reads using the default settings [25]. BBmask was used to soft-mask human, cat, dog, and mouse contamination. MEGAHIT (version 1.2.8) was then used for de novo assembly into contigs [26]. VirSorter2 (version 2.2.2) was then used to identify putative viral contigs (<0.22 µm fraction) [27,28]. All identified contigs were kept for further analyses, and these contigs were confirmed to be host-free as they all returned no SSU rRNA sequences when screened with PhyloFlash (version 2.0) [29]. Gene prediction was made with Prodigal (version 2.6.3) [30]. Genes and proteins were annotated with DIAMOND (version 0.9.26), utilizing the “sensitive” setting and NCBI protein non-redundant database (created on 23 August 2019) [31]. Viral assemblage analysis and diversity measures were calculated in MEGAN [32] with the R package vegan [33]. vCONTACT2 [34] was used to compare viral contigs from Stn7 and construct a network visualized with Cytoscape [35]. We used the number of viral contigs identified in the microbial fraction (>0.22 µm) as a proxy to estimate the frequency of viral infections. To do so, VirSorter was used to identify putative viral contigs in the >0.22 µm fraction metagenomes. For putative lytic infections, categories 1 and 2 were kept, while for the putative prophages, categories 4 and 5 were kept.

Environmental variables were normalized and put on the same unitless scale utilizing a z-score transformation. Viral families were identified in MEGAN and square root transformed before being plotted on the non-metric multidimensional scaling plot (NMDS). The NMDS was plotted with the R packages vegan and ggplot2 [36]. Due to the lack of complete metadata for the pre-Harvey samples, the NMDS only includes the post-storm samples. Diversity analysis (Shannon index, Pielou index, and ACE index) of viral metagenomes was done in R-Studio version 4.0.3 with packages from fossil version 0.4.0 [37], vegan version 2.5.7 [33], cluster version 2.1.2 [38], gplots version 3.1.1 [39], pvclust version 2.2.0 [40], metagenomeSeq version 1.32.0 [41], and RCurl version 1.98.1.5 [42]. Analysis was done, and figures were made following an R script previously published [43].

### 2.4. Identification of Putative AMGs 

VIBRANT (version 1.2.0) was used to identify and annotate viral contigs in microbial (>0.22 µm) and viral (<0.22) metagenomes utilizing the -virome argument and default parameters (1000 bp sequence input and 4 ORFs per scaffold) and to find potential metabolic genes, or AMGs [44]. AMGs are identified in VIBRANT by assigning functional genes to KEGG metabolic pathway maps and then further sorting into broader KEGG annotation categories. In this study, we kept only metabolic genes involved in energy and carbohydrate metabolisms that have been identified as AMGs in other studies. AMG counts were normalized to one million proteins, and heat maps were built in R Studio with ggplot2 [36].

### 2.5. Viral Abundance

Viral-like particles were enumerated from all the Stn7 samples and the 22 August pre-Harvey sample using epifluorescence microscopy as described in [45]. Briefly, 100 µL of virus concentrate was fixed with 37% paraformaldehyde before filtration on a 0.02 µm Anodiscs (Whatman, Marlborough, MA, USA). Filters were stained with 1:400 SYBR Green I, and 10% (*w/v*) *p*-phenylenediamine dihydrochloride was used as an anti-fade. At least 20 fields of view were counted, and the average was used to calculate the concentration per ml of virus concentrate. Viral abundance in the original sample was estimated based on the concentration factor. It is worth noting that the concentrations are only estimates because there may be loss during tangential flow filtration virus concentration [46,47], and we did not have preserved samples from the original samplings.

### 2.6. Phylogenetic Analysis

All gokushovirus genomes available in NCBI and viral contigs sharing similarity with gokushoviruses (Clusters 2 and 3) were annotated using RAST [48]. Genes annotated as “Phage major capsid protein #Fam0039” and “Phage major capsid protein” were translated into proteins and aligned using MAFFT with the E-INS-I algorithm [49,50]. Sequences containing the N-terminal half (as in [51]) were kept for further analysis. Maximum likelihood phylogenetic analysis was performed in MEGA11 [52] with the LG + I + F model as determined within MEGA11. Trees were viewed in FigTree (https://github.com/rambaut/figtree/ (accessed on 16 November 2021)).

### 2.7. Data Availability

All metagenomes are publicly available in the MG-RAST metagenomics analysis server [53] and NCBI SRA Archive (accession numbers listed in Supplementary Table S3).

## 3. Results and Discussion

Models predict that rainfall rates associated with tropical cyclones will increase by 10–15% in the coming years, and these cyclones will intensify [54]. With viruses playing a vital role in marine geochemical cycles by recycling organic nutrients via the lysis of plankton in pelagic [9,10,55] and coastal environments [56,57], it is important to understand how viruses are impacted by such disturbances and the consequences of that impact. Two other studies evaluated the impacts of heavy rainfall on viral communities and showed that stormwater runoff can significantly change the composition of the viral assemblages in freshwater retention ponds [19,20]. In this study, we provide one of the first looks at how heavy rainfall events can impact marine viral assemblages, both at the taxonomic and functional level, in estuarine environments. Utilizing Hurricane Harvey as a pulse disturbance, we can evaluate how the viruses in other coastal bays similar in structure to Galveston Bay (i.e., riverine input, hurricane-prone, coastal locations), such as those located along the Gulf of Mexico coast, could be impacted by large rain events. 

### 3.1. Hurricane Harvey Changed the Viral Community Diversity and Composition

We sequenced viral metagenomes (<0.22 µm fraction) from four samplings over the five weeks following Hurricane Harvey along a four-station transect across Galveston Bay, from the San Jacinto River to the mouth of the Gulf of Mexico (Figure 1 and Supplementary Table S2). We also sequenced the viral metagenomes from two pre-Harvey samples located nearby Stn7. On average, each metagenome consisted of ~37 million reads, which resulted in an average of ~169 thousand contigs with an N50 length of 944 bp and ~38 thousand proteins (Supplementary Table S3).

**Figure 2:**
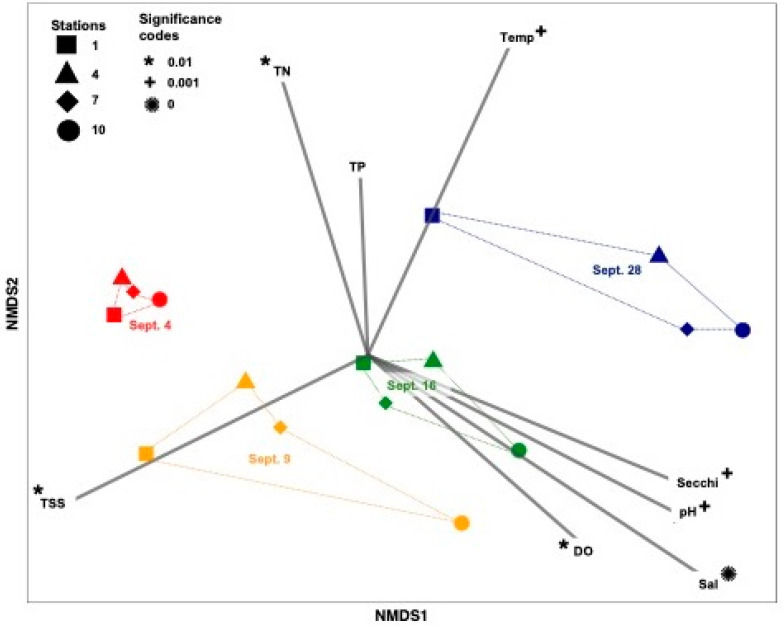
NMDS of the viral community composition showing the correlation between temperature (Temp; °C), salinity (Sal), pH, dissolved oxygen (DO; %), total nitrogen (TN; mM), total phosphorus (TP; mM), Secchi (m), and total suspended sediment (TSS; g mL^−1^). All data for environmental variables were taken from [17]. A “*” represents a significance level of *p* = 0.01. Colors represent sampling time points in 2017, and symbols represent sampling stations. NMDS was constructed using viral metagenomic abundances at the family level using a square root transformation and a Bray-Curtis similarity matrix, with a stress level of 0.0510.

Previous studies have shown that Hurricane Harvey dramatically changed Galveston Bay. The heavy rainfall, stormwater runoff, and sediment resuspension contributed to decreasing salinity, pH, and temperature and increasing total resuspended sediment, turbidity, and total nitrogen in the system [17,21,22]. A non-metric multidimensional scaling (NMDS) analysis revealed clear temporal differences between samples taken at each sampling date (Figure 2) as stations grouped together by sampling date (Figure 2). We identified correlations between environmental variables and the viral composition to determine which factor was potentially driving the changes. Salinity was the environmental driver with the strongest impact (*p* < 0.001) on the viral assemblages, followed by temperature, turbidity (Secchi depth), and pH (*p* < 0.05) (Figure 2). Total suspended sediment (TSS), total nitrogen, and dissolved oxygen had no significant impact on the viral assemblages (*p* > 0.1) (Figure 2). The diversity of viruses in the ecosystem decreased after the rain event, which is the opposite of what was observed for the microbial community (Walker et al., unpublished) [17].

The drastic environmental changes caused by Hurricane Harvey changed the viral richness and diversity in the samples (Table 1 and Supplementary Figure S1). The Shannon diversity index calculations revealed that 4 September had the lowest richness (ACE index) and evenness (Pielou index) among the post-storm samples (Table 1). This suggests an uneven distribution of the taxa with a few dominant members. Viral diversity and richness then gradually increased throughout the study (Table 1). One hypothesis for the lower diversity is that the record amount of rainfall possibly diluted the viruses. To test whether the change in richness and evenness was the result of a potential dilution of the viruses following the rain, we counted the number of virus-like particles in each virus concentrate and estimated viral abundance in the original sample based on the concentration factor. If we assume that each sample had similar viral recovery and decay prior to counting, we observe fewer viruses after the storm, which could be due to the large input of rainwater that diluted the viruses (Supplementary Figure S2). Another explanation for the lower abundance of viral particles after the storm is that the inflow of water and new viruses reduced the virus–host contact rate, reducing viral infections and, therefore, viral production. It is also possible that viral concentrations were reduced due to changes in environmental parameters such as the change in temperature, presence of pollutants, or grazing that may have increased viral degradation [58]. 

**Figure 3:**
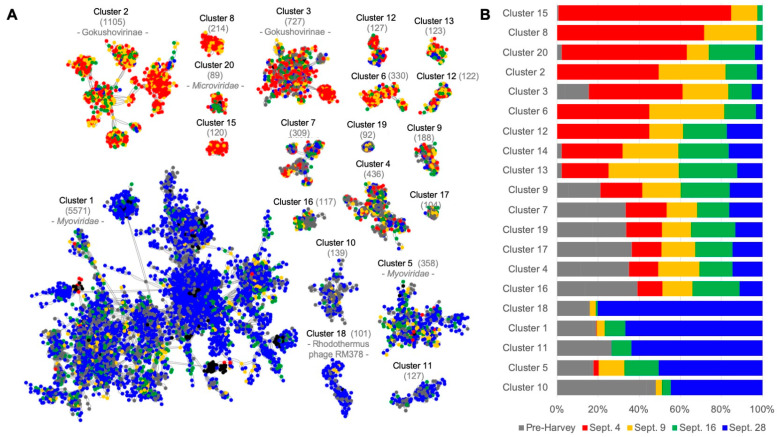
Comparison of the viral contigs to each other at Stn7. (**A**) Gene-sharing network of viral contigs generated with vConTACT v2.0 and visualized using an edge-weighted spring-embedded algorithm that locates more similar contigs closer to each other. Nodes represent contigs from 4 September (red), 9 September (yellow), 16 September (green), 28 September (blue), and pre-Harvey (grey). Clusters were named based on the number of contigs in each, 1 being the cluster with the most contigs. The number of nodes in each cluster is indicated in parentheses, and the identification of the reference genomes is indicated when available. (**B**) Relative abundance of the number of contigs from each sampling date in each identified cluster, ordered by the abundance of 4 September contigs (decreasing) and 28 September contigs (increasing).

The viral assemblages’ taxonomic composition at the family level revealed drastic, short-term changes after Hurricane Harvey. Viral contigs were first compared and grouped into clusters (Figure 3). Most clusters had no similarity to reference genomes unless otherwise stated. Five of the 20 largest clusters, Clusters 15, 8, 2 (related to *Gokushovirinae*), 6, and 12, had no similarity with the viruses found prior to Hurricane Harvey (Figure 3), suggesting that the storm brought novel viruses into the system. Three viral groups, Clusters 11, 18 (related to *Myoviridae* Rhodothermus phage RM378), and 10, present before the storm, were absent on 4 September, and their abundances gradually increased as the environmental conditions returned to estuarine conditions similar to pre-Harvey (Figure 3). The group with the most contigs, Cluster 1 (sharing similarities to many Cyanophages, *Pelagibacter* phages, and other *Caudauvirales* infecting marine microorganisms), was predominantly composed of contigs from 28 September and pre-Harvey metagenomes. The re-emergence of Cluster 1 on 28 September suggests the viral community was returning to that of an estuarine environment.

**Figure 4:**
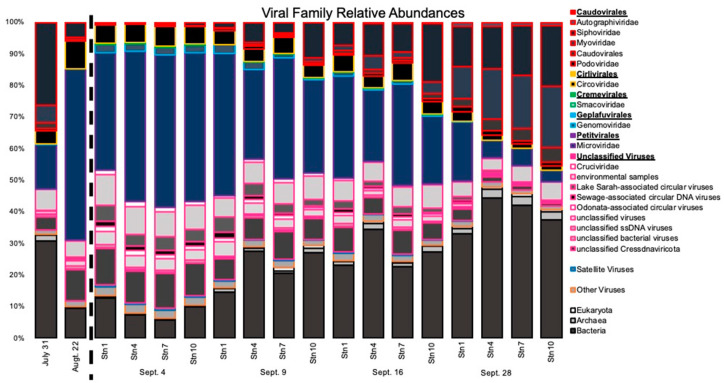
Stacked bar histogram showing the relative abundance of viral families identified by metagenomic viral DNA samples. Sampling points are broken up into blocks separated by white space. The dotted line separates pre- and post-Harvey samples.

**Figure 5:**
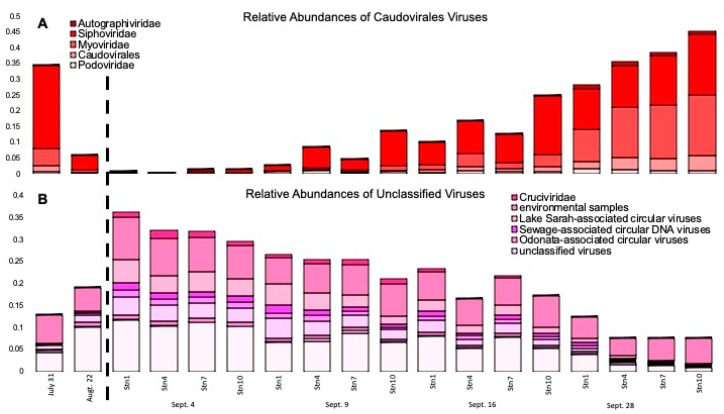
Stacked bar histogram showing the relative abundance of the viral family (**A**) Caudovirales and (**B**) Unclassified viruses. Both families were identified by metagenomic viral DNA samples. The dotted line separates pre- and post-Harvey samples.

We then compared the viral assemblages to the NCBI nr database to assign taxonomy. Taxonomic classification of the viral proteins also revealed a change in the viral composition immediately after the storm (Figure 4). The family *Siphoviridae* was the most abundant viral family in July, prior to Hurricane Harvey’s landfall, with a relative abundance of 26% of the total viral assemblages. Relative abundances of *Siphoviridae* fell to an average relative abundance of 0.54% on 4 September (Figure 4 and Figure 5). Relative abundances of the family *Microviridae* increased almost three-fold following Hurricane Harvey, from an average relative abundance of 14.13% of the total viral assemblage pre-Hurricane Harvey to 45.16% on 4 September (Figure 4). Following Hurricane Harvey’s landfall, proteins representing the viral families *Siphoviridae*, *Podoviridae,* and *Myoviridae* almost disappeared from Galveston Bay but returned by 28 September. There was also an influx of proteins associated with freshwater invertebrate viruses grouped into the category Lake Sarah Associated Circular Viruses (Figure 5), a group of freshwater viruses assembled from metagenomes recovered from a New Zealand lake, Lake Sarah [59]. These freshwater viruses were very low in abundance in the viral assemblages prior to Hurricane Harvey (0.32% of the total assemblage); however, on 4 September, the proteins from this group comprised 4.40% of the total viral assemblage. The average relative abundance of proteins associated with these freshwater viruses had receded to 0.58% of the total viral assemblage by 28 September (Figure 5).

The decrease in the relative abundance of *Podoviridae* and *Myoviridae* and the dominance of a single family, the *Microviridae*, could be responsible for the changes in richness and diversity. *Podoviridae* and *Myoviridae* are commonly the most abundant viral families in coastal environments [8]. They were also the most abundant families prior to the storm and in our last sampling when environmental conditions were back to estuarine conditions. Most known Cyanophages belong to the *Podoviridae* or *Myoviridae* families [60,61]. Besides Cyanobacteria, *Podoviridae* are also known to infect common marine bacteria such as *Pelagibacter* [62]. These findings are comparable to how stormwater runoff rapidly changes freshwater viral communities and is a driver in community composition [19].

While *Microviridae*, especially *Gokushovirinae* (small circular genomes of <5.5 kb)*,* are present in our Galveston Bay samples, their relative abundances may not be accurate due to the polymerase used for whole genome amplification that has a template bias for small circular genomes, resulting in a higher number of reads associated with these genomes [63,64,65,66]. Since we had to resort to whole genome amplification to get enough DNA for metagenomic sequencing, it is possible that members of the *Microviridae* family were over-amplified. It has been well documented that gokushoviruses are widely distributed throughout the marine environment [67,68]. The widespread nature of these viruses in the marine environment makes it difficult to attribute the increase in gokushoviruses after Hurricane Harvey to the storm. We performed a phylogenetic analysis of the major capsid protein gene to look at the genetic relatedness of the gokushoviruses from this study and other environments and clinical isolates (Supplementary Figure S3). Our analysis shows that the gokushoviruses from Galveston Bay are distinct from the isolates, which are phages of *Chlamydia* and *E.coli*, and distinct from most gokushoviruses isolated from animal and human guts. One cluster contained sequences observed only after Hurricane Harvey hit, suggesting that these are of terrestrial and freshwater origin and were brought into the system by the storm. Our results confirm that gokushoviruses are diverse and ubiquitous but occupy specialized niches [67].

### 3.2. The Number of Viruses Identified in the Microbial Fraction Escalated throughout the Weeks Following Hurricane Harvey

We identified the viral contigs from lytic infections and prophages in the microbial metagenomes (>0.22 µm; Table 2). The lowest number of viral contigs identified in the microbial metagenomes were observed prior to and immediately after Hurricane Harvey. Over time, the abundance of lytic viruses increased, and by 16 September, they were 3.6× more abundant than on 4 September or prior to the storm. The presence of viral contigs in the microbial fraction can indicate ongoing infections [69], ingested viruses [70], or viruses that attached to cells, particles, or filters during the filtration process. Here we used the abundance of viral contigs from lytic infections as a proxy to estimate the relative abundance of ongoing viral infections. To do so, we assume that active viruses should be more abundant than particle-associated viruses due to the high burst size of marine heterotrophic bacterial infections in marine environments (average burst size of ~24–34 viruses [71]). Moreover, there were more resuspended particles in the earlier samplings after the storm, yet the number of identified viral contigs was lower. The low incidence of lytic infections following the storm is likely the result of the heavy rainfall that reduced the virus–host encounter rate and created a disconnection between viruses and their hosts. After Hurricane Harvey, with the virus–host contact rate reduced, the novel microbial hosts introduced into the system from stormwater runoff and sediment were able to thrive. Once host abundances became high enough to increase the contact rate, viral infections increased, behaving in a “Kill-The-Winner” fashion [72]. An increase in lytic infections will likely increase the viral shunt, accelerating nutrient recycling [8,11]. It has been documented that Hurricane Harvey altered carbon exchange in the marine environment [73]. The contribution of viruses to this increase in the carbon cycle may be greater than previously thought and may have long-term effects on the ecosystem. 

The abundance of identified prophages also increased after Hurricane Harvey. The increase started on 4 September for Stns 7 and 10, increased leading to 9 September, and peaked on 16 September. Only the freshwater stations had prophage-associated sequences by 28 September. No metabolic genes were identified within the prophages, which is probably due to the low numbers of identified prophages (536 total contigs) compared to the total number of contigs in the viral metagenomes (160,999 contigs). However, an increased incidence of prophages could be linked to the increase in observed metabolic genes. Indeed, viruses have been shown to act as a great source of available functions that can be transferred to their hosts via transduction [2].

We taxonomically classified the viral proteins identified within the microbial metagenomes (Supplementary Figures S4 and S5). For the lytic viruses, a majority of the proteins were classified as cellular organisms or as members of the order Caudovirales (Supplementary Figure S4). Members of the order Caudovirales contain some of the most abundant viruses identified in the ocean [8,74] and infected cells [75]. The taxonomic distribution differed greatly from the viral proteins identified in the viral metagenomes, which was most likely due to the small number of contigs identified and the use of whole-genome amplification on the viral DNA, which skews viral concentrations. However, the identification of single-stranded DNA viruses in the microbial metagenomes, which have not been subjected to whole genome amplification, confirms that the sequences identified in the viral metagenomes are not artifacts. As for the prophages, after the storm, most of the prophages carried a majority of genes related to their bacterial hosts (i.e., Verrucomicrobia, Comamonadaceae, Rhodospirillaceae, and Rhodobacteraceae), which were related to the terrestrial and freshwater microbes brought in by the flood and storm waters [17]. In the later samplings, the prophages carried, almost exclusively, genes related to the order Caudovirales.

### 3.3. Hurricane Harvey Increased the Abundance of Virally Encoded Metabolic Genes Involved in Major Biogeochemical Cycles

**Figure 6:**
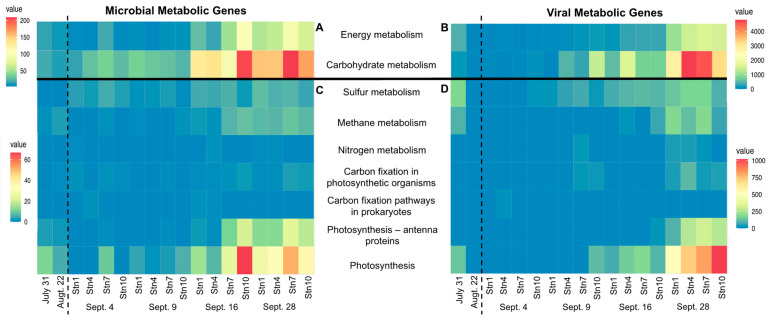
Heat map showing the density of metabolic genes, normalized to one million proteins, recovered from microbial metagenomes (A, C) and viral metagenomes (B, D). A and B show a breakdown of metabolic genes associated with all metabolism pathways in microbial and viral metagenomes, respectively. C and D show a breakdown of metabolic genes associated with energy metabolisms in microbial and viral metagenomes, respectively. In the viral metagenomes on 4 September at Stn1 and Stn7 and on 22 August, no metabolic genes were found. The dotted line separates pre- and post-Harvey samples.

To determine the potential impact of viral infections on the geochemical cycles of Galveston Bay, we searched for metabolic genes in the viral (<0.22 µm) and microbial (>0.8–0.22 µm) metagenomes. The density of metabolic genes was more than one order of magnitude more in viral metagenomes than in microbial metagenomes. While the metabolic gene concentration was higher in viruses, their detection was delayed by one sampling for each station (e.g., metabolic genes involved in energy metabolisms were identified first on 16 September and peaked on 28 September in the microbial fraction, but only on 28 September for viruses). Metabolic genes associated with the metabolisms of cofactors and vitamins, amino acids, and carbohydrates were the most abundant (Figure 6). The abundance of metabolic genes associated with energy metabolism follows a similar trend to the ones found in the microbial fraction. The metabolic genes involved in energy and carbohydrates were barely detectable prior to Hurricane Harvey, then increased over time to display the highest abundances in the later samples, five weeks following the storm (A in Figure 6). While the microbial fraction displayed the highest concentrations of metabolic genes on 16 September at Stn4 and Stn7, and on 28 September, at all of the stations, viruses were higher only on 28 September, suggesting that the viral cycle is delayed compared to the microbial fraction or their putative host population. This trend is most obvious with carbohydrate metabolism, which includes pathways associated with glycolysis, pentose phosphate, and amino and nucleotide sugar metabolism (Supplementary Figure S6). Metabolic genes associated with energy metabolism, such as photosynthesis, methane, and sulfur, dropped after Hurricane Harvey’s landfall (A and B in Figure 6). Metabolic genes associated with sulfur metabolism were the first to appear consistently in the viral metagenomes on 9 September at Stn10 (B in Figure 6), which is congruent with the sulfur metabolic genes observed in the microbial metagenomes in all stations on 9 September (A in Figure 6).

Viral AMGs reflecting host metabolisms and adaptations have previously been documented in different datasets, including in the Pacific Ocean Virome database, where it was shown that viral AMGs are depth-stratified based on the host distribution [76]. A similar trend where viruses follow the cellular population distribution can also be observed in our dataset, especially when looking at the genes associated with photosynthesis (A and B in Figure 6); therefore, we hypothesize that our metabolic genes are, in fact, auxiliary metabolic genes (AMGs) although we are unable to confirm. Photosynthetic microbes, such as Cyanobacteria, were depleted from Galveston Bay following Hurricane Harvey [17]. Viral metabolic genes associated with the photosynthetic pathway were detected only once the microbial community, mainly consisting of Cyanobacteria, had recovered on and after 16 September [17] (B in Figure 6). The loss of primary producers is also corroborated with a decrease in surface chlorophyll *a* following Hurricane Harvey in other coastal Texas bays [22].

There was a slight increase in metabolic genes associated with sulfur metabolisms following Hurricane Harvey (B in Figure 6). Genes in this pathway are similar in function to *cysP*, which binds to thiosulfate and aids in transmembrane transport [77]. This increase in metabolic genes associated with sulfur metabolisms is hypothesized to be linked to the sediment resuspension seen in Galveston Bay following Hurricane Harvey. The resuspension of sediment is confirmed by the highest concentrations of total suspended sediment occurring during time points following Hurricane Harvey’s landfall [17] and documented heavy sediment loading [21]. A significant part of the microbial community within marine sediment utilizes sulfur compounds as an energy source [78]. Metabolic genes associated with sulfur metabolisms have previously been observed in other environments, including the epipelagic zone [7] and the deep-sea [79], suggesting that viruses may play an important role in sulfur cycling.

Within the identified pathways associated with carbohydrate metabolism, five had obvious changes following Hurricane Harvey’s landfall: the pentose phosphate pathway, pentose and glucuronate interconversions, galactose metabolism, fructose and mannose metabolisms, and amino and nucleotide sugar metabolisms (Supplementary Figure S6). Genes represented in these metabolism pathways include *pgi*, *galM,* and *mak*, which are involved in catalytic activity; *kduI,* which substitutes the normal hexuronate degrading enzyme under osmotic stress; and *nagZ,* which is involved in peptidoglycan recycling [80]. Hurricane Harvey floodwaters caused a large influx of dissolved organic carbon (DOC) within Galveston Bay [17,73]. The influx of DOC to the bay from the floodwaters could be the cause of the increase in metabolic genes associated with carbohydrate metabolisms (Supplementary Figure S6). Previous work done in the Arctic Ocean found that a decrease in total dissolved carbohydrates accounted for half of the DOC decrease seen within the upper 200 m of the ocean [81], linking carbohydrates and DOC. Similarly, an increase in DOC in Galveston Bay could cause an increase in carbohydrates, which would account for the increase in heterotrophs and microorganisms able to degrade carbohydrates. Since virally encoded metabolic genes are typically also found within their hosts [3], the infection of these microorganisms could lead to an increase in viruses carrying metabolic genes from carbohydrate degradation pathways. Moreover, Hurricane Harvey caused an acceleration of the DOC cycle in Galveston Bay via higher rates of mineralization of terrigenous DOC, which was linked to the active microbial community [73]. Our results show that this acceleration could be exacerbated by viruses, as is shown by the increase in viral metabolic genes associated with carbohydrate metabolisms, which are orders of magnitude higher post-Hurricane Harvey compared to the pre-storm samples (Supplementary Figure S6).

## 4. Conclusions

It is projected that rainfall rates associated with tropical cyclones will increase in the coming years due to climate changes [54], making these intense rainfall events more common. It is important to understand how viral assemblages, which are linked to their hosts, respond to intense rain events. We provided the first in-depth look at marine viral assemblages’ impact and recovery from intense rainfall. Immediately after the storm, viral communities were altered by the introduction of novel taxa, a decreased viral abundance, and a decreased frequency of viral infections, all of which resulted in lower viral diversity. There was an increase in AMGs associated with carbon, nitrogen, and sulfur metabolisms that may have aided the host community in the unfavorable environmental conditions brought by the storm; highlighting how viruses can play an important role in ecosystem recovery from large scale rain events or other pulse disturbances. After five weeks, there was greater representation of virus-associated sequences in the microbial fraction than prior to or just after the storm, suggesting that the impact of the storm on the geochemical cycles in the long term may be more important than previously thought. Future studies should quantify how the viral load is impacted by rainfall to further understand the impact of storms on viruses and their role in ecosystem recovery. 

## Figures and Tables

**Table 1:** Diversity (Shannon), evenness (Pielou), and richness (ACE) indices of the viral metagenomes at the family level.

Dates in 2017	Station	Shannon Index	Pielou Index	ACE Index
31 July	TAMUG Boat Basin	2.01	0.59	33.73
22 August	TAMUG Boat Basin	1.57	0.45	36.91
4 September	Stn1	1.93	0.55	34.45
	Stn4	1.73	0.49	36.01
	Stn7	1.72	0.49	35.13
	Stn10	1.70	0.47	37.08
9 September	Stn1	1.86	0.51	38.47
	Stn4	2.16	0.60	37.69
	Stn7	1.92	0.54	37.44
	Stn10	2.07	0.58	36.43
16 September	Stn1	2.08	0.58	36.21
	Stn4	2.22	0.62	37.46
	Stn7	2.09	0.58	37.42
	Stn10	2.14	0.60	35.00
28 September	Stn1	2.23	0.62	38.25
	Stn4	2.08	0.58	40.54
	Stn7	1.96	0.55	40.75
	Stn10	1.85	0.53	40.51

**Table 2:** Total number of lytic viruses and prophages, normalized to 10,000 contigs, recovered from microbial metagenomes.

Dates in 2017	Station	Lytic Viruses	Prophages
4 September	Stn1	28.7	0.0
	Stn4	44.6	0.0
	Stn7	69.2	0.5
	Stn10	47.8	3.0
9 September	Stn1	58.6	0.6
	Stn4	61.3	1.9
	Stn7	62.0	1.6
	Stn10	37.5	1.6
16 September	Stn1	147.1	1.1
	Stn4	152.7	5.0
	Stn7	116.8	2.6
	Stn10	199.6	0.6
28 September	Stn1	184.8	2.2
	Stn4	165.3	4.6
	Stn7	199.5	0.0
	Stn10	144.0	0.2
31 July	Pre-Harvey1	39.5	0.0
22 August	Pre-Harvey2	28.5	0.0

## Data Availability

All metagenomes are publicly available in the MG-RAST metagenomics analysis server [53] and NCBI SRA Archive (accession numbers listed in Supplementary Table S3).

## Supplementary Materials

The following supporting information can be downloaded at https://www.mdpi.com/article/10.3390/microorganisms10040729/s1, Supplementary figure and Tables (docx); Table S1: Metadata from Steichen et al. showing a drop in salinity, temperature, and dissolved oxygen following Hurricane Harvey; Table S2: Types of filters used and volume filtered for each sample; Table S3: Reads and contigs statistics and accession numbers for each sequenced metagenome. Figure S1: Estimates of viral diversity (Shannon index), evenness (Pielou index) and richness (ACE index) from the viral metagenomes of A) pre-Harvey samples (July 31 & Augt. 22) compared to the first sampling (Sept. 4) and B) Sept. 4 sampling, compared to the last sampling effort (Sept. 28).; Figure S2: Virus-like particles abundances (with standard error bars)in samples from Stn7 and the Augt.22 pre-storm sample. Counts were performed on the viral concentrates and the total for the original samples are estimated based on the concentration factor, assuming a 100% concentration efficiency.; Figure S3: Unrooted phylogenetic analysis (maximum likelihood; LG + I + F model; 100 bootstrap replicates) of the gokushovirus major capsid protein (Sept.4 in red, Sept.9 in orange, Sept.16 in green, and Sept.28 in blue, pre-Hurricane. Harvey in grey, and isolates in black). Bootstrap support is represented by a gradient from white (0% support) to black (100% support). The large black clade solely contains sequences from isolates from animal guts. The highlighted grey clade contains sequences from post-Hurricane Harvey only.; Figure S4: Stacked bar histogram showing the relative abundance of the lytic viruses identified in the host metagenomes. The dotted line separates Pre- and Post-Harvey samples. Figure S5: Stacked bar histogram showing the relative abundance of the prophages identified in the host metagenomes. The dotted line separates Pre- and Post-Harvey samples.; Figure S6: Heat map showing the density of auxiliary metabolic genes (AMGs) related to carbohydrate metabolisms, normalized to one million proteins, recovered from microbial metagenomes (A) and viral metagenomes (B). July 31 and Augt. 22 were sampled prior to Hurricane Harvey’s landfall.
