# Supplementary material for: Record-Breaking Rain Event Altered Estuarine Viral Assemblages
Source: Microorganisms. 2022 Mar 29;10(4):729. doi: 10.3390/microorganisms10040729 (PMC9025952; doi:10.3390/microorganisms10040729)
Supplement: Supplementary file 1 [file microorganisms-10-00729-s001.zip › viral_community_Supplemenrary_Figures.pdf]

Supplementary Figures

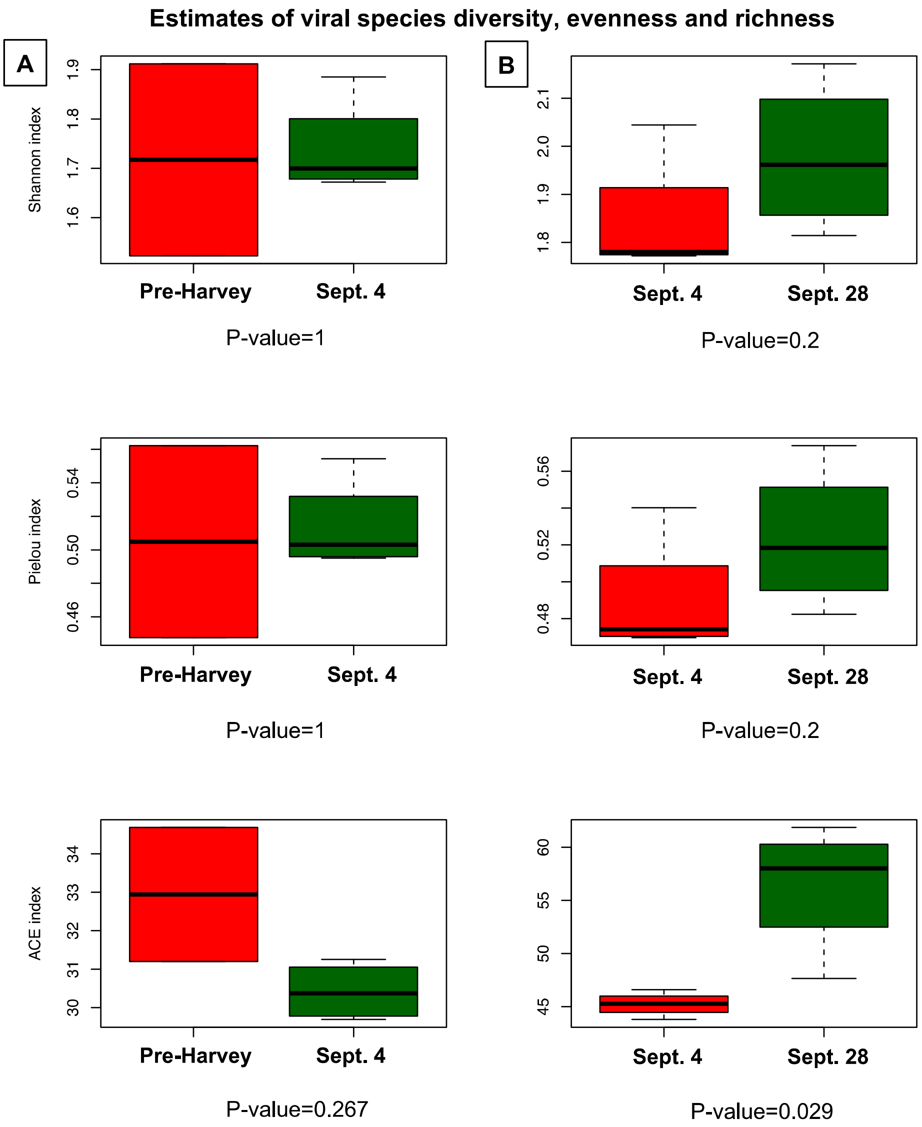

Figure S1. Estimates of viral diversity (Shannon index), evenness (Pielou index) and richness (ACE index) from the viral metagenomes of (A) pre-Harvey samples (July 31 & Augt. 22) compared to the first sampling (Sept. 4) and (B) Sept. 4 sampling, compared to the last sampling effort (Sept. 28).

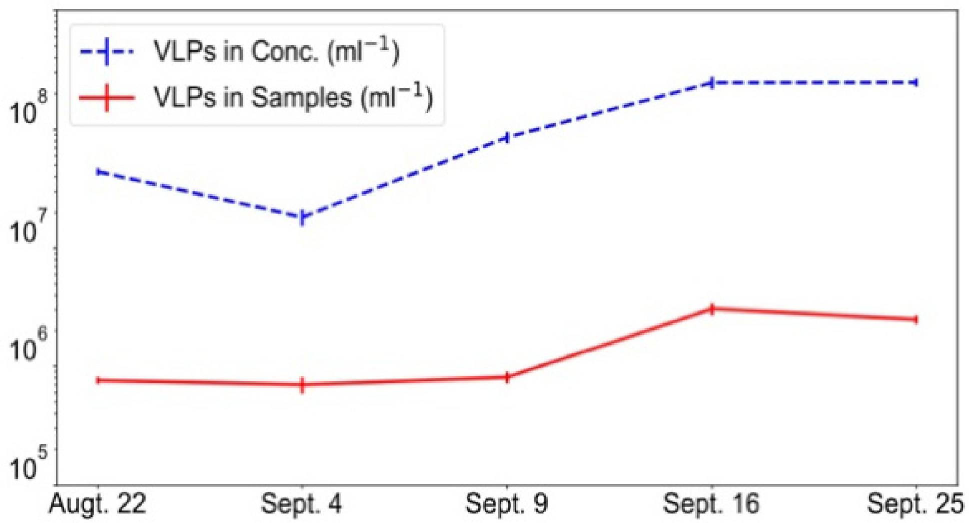

Figure S2. Virus-like particles abundances (with standard error bars)in samples from Stn7 and the Augt. 22 pre-storm sample. Counts were performed on the viral concentrates and the total for the original samples are estimated based on the concentration factor, assuming a 100% concentration efficiency.

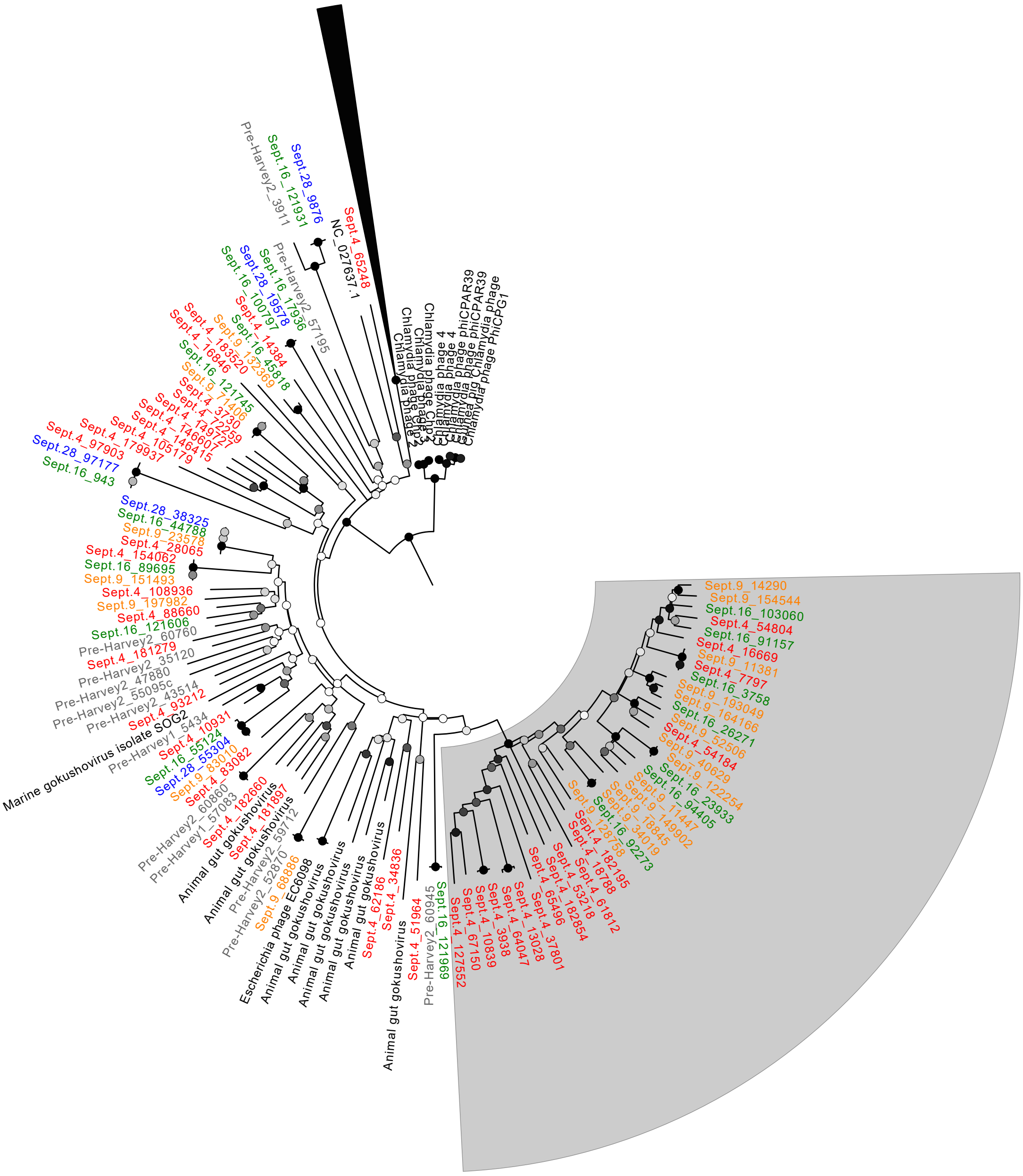

Figure S3. Unrooted phylogenetic analysis (maximum likelihood; LG + I + F model; 100 bootstrap replicates) of the gokushovirus major capsid protein (Sept. 4 in red, Sept. 9 in orange, Sept. 16 in green, and Sept. 28 in blue, pre-Hurricane. Harvey in grey, and isolates in black). Bootstrap support is represented by a gradient from white (0% support) to black (100% support). The large black clade solely contains sequences from isolates from animal guts. The highlighted grey clade contains sequences from post-Hurricane Harvey only.

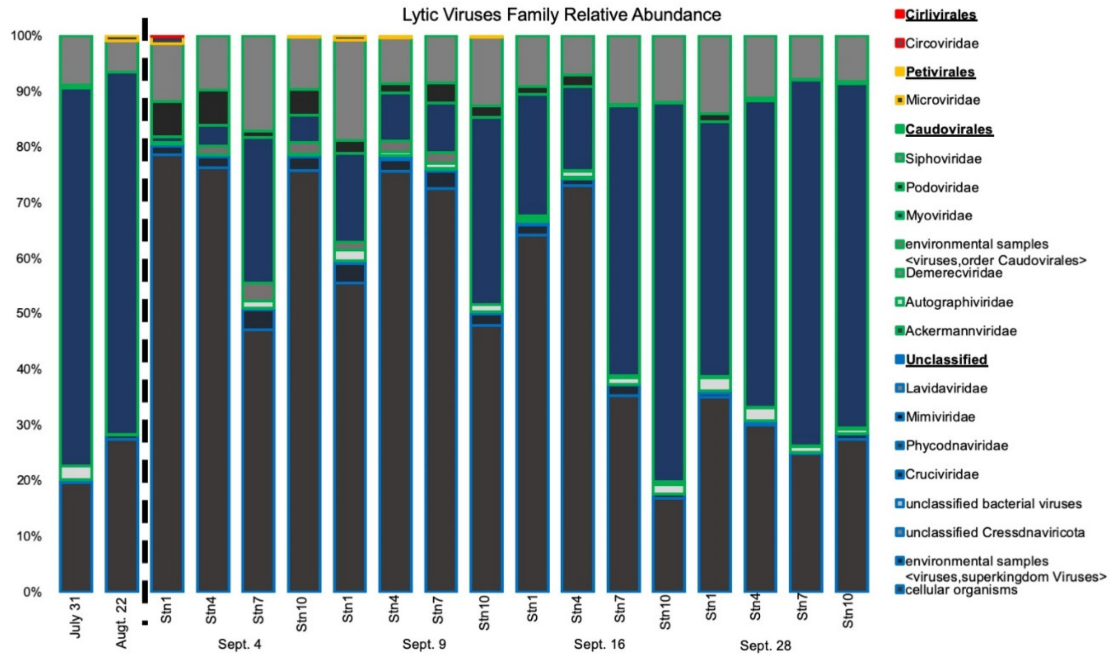

Figure S4. Stacked bar histogram showing the relative abundance of the lytic viruses identified in the host metagenomes. The dotted line separates Pre- and Post-Harvey samples.

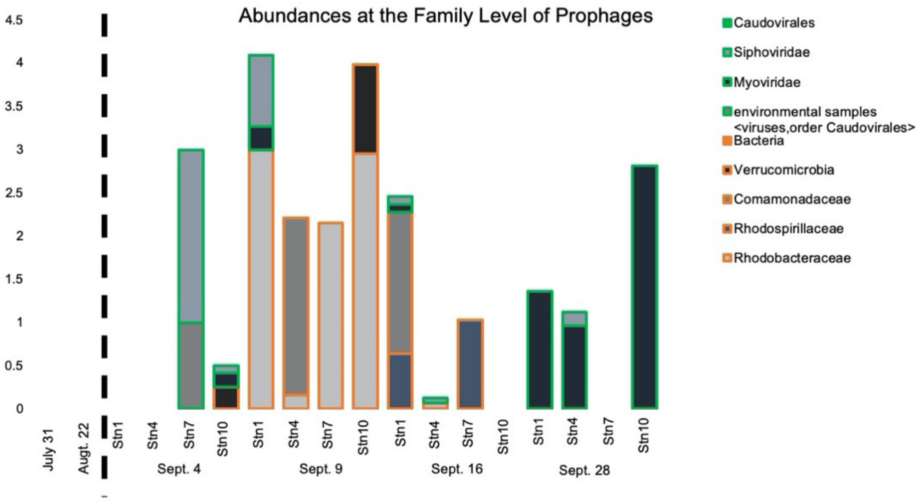

Figure S5. Stacked bar histogram showing the relative abundance of the prophages identified in the host metagenomes. The dotted line separates Pre- and Post-Harvey samples.

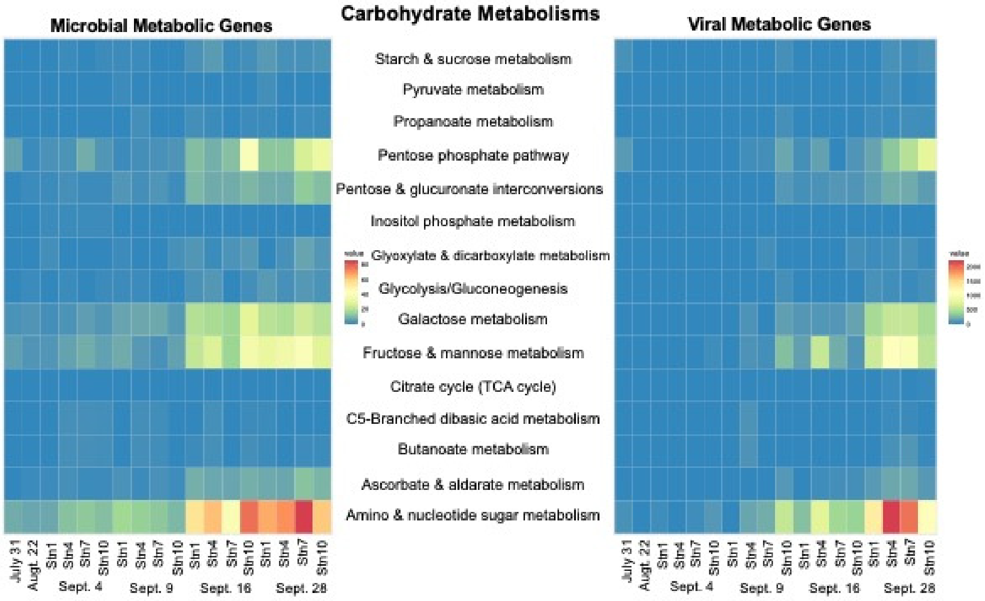

Figure S6. Heat map showing the density of auxiliary metabolic genes (AMGs) related to carbohydrate metabolisms, normalized to one million proteins, recovered from microbial metagenomes (A) and viral metagenomes (B). July 31 and Augt. 22 were sampled prior to Hurricane Harvey's landfall.
